# Na^+^/K^+^ Hybrid Battery Based on a Sulfurized Polyacrylonitrile Cathode

**DOI:** 10.3390/ma12060969

**Published:** 2019-03-23

**Authors:** Jin Lou, Youqiang Zhang, Yi Shuai, Kanghua Chen, Songyi Chen

**Affiliations:** 1 Light Alloy Research Institute, Central South University, Changsha 410083, China; jinlou@csu.edu.cn (J.L.); shuaiyi@csu.edu.cn (Y.S.); sychen08@csu.edu.cn (S.C.); 2 Collaborative Innovation Center of Advance Nonferrous Structural Materials and Manufacturing, Central South University, Changsha 410083, China; 3 State Key Laboratory of High Performance Complex Manufactring, Central South University, Changsha 410083, China; youqiangzhang@csu.edu.cn; 4 Powder Metallurgy Research Institute, Central South University, Changsha 410083, China

**Keywords:** Na^+^/K^+^ hybrid battery, suppressed dendrite, sulfurized polyacrylonitrile

## Abstract

Sulfurized polyacrylonitrile (SPAN) nanocomposites were synthesized and used as a cathode in a novel rechargeable Na^+^/K^+^ hybrid battery with high performance for the first time. When 0.9 mol NaPF_6_ and 0.1 mol KPF_6_ were dissolved in ethylene carbonate (EC)/dimethyl carbonate(DMC)/ethyl methyl cabonate(EMC) (4:3:2, v/v/v), used as hybrid electrolyte, Na foil was used as the anode, and SPAN composites were used as the cathode, a hybrid ion system was created via composition–decomposition between Na^+^/K^+^ and SPAN and stripping–depositing of Na^+^ with suppressed dendrites by taking advantage of the self-healing electrostatic shield effect. As a result, a highly reversible calculated capacity of 1405.5 mAh g_sulfur_^−1^ with a coulombic efficiency approaching 100% after 100 cycles was obtained at a current density of 35 mA g^−1^. This environmentally benign, low-cost Na^+^/K^+^ hybrid battery shows promise as a new future flexible energy storage system (ESS) technology.

## 1. Introduction

For decades, lithium-ion batteries (LIBs) have been the dominant energy storage systems (ESSs) due to their high theoretical specific energy and technological advancements [1,2]. Among recent progress in LIBs research, lithium–sulfur (Li–S) batteries are being considered as next-generation flexible ESSs because of their high energy density, inherent safety, and low cost [3,4,5]. In Li–S battery research, polymers, including polypyrrole (PPy), polyaniline (PANI), and polyacrylonitrile (PAN), have been investigated as electroactive cathode materials [6]. Because of its stable electrochemical performance and durability, the mechanism of Li-sulfurized PAN (SPAN) storage has been studied in detail [7,8,9]. However, research on LIBs is approaching the theoretical energy density limit. As the ubiquitous demand for portable devices and large-scale applications drastically increases, the rising price of Li further promotes the need to explore alternative, effective, and high-energy-density storage systems [10,11,12]. The earth’s crust contains sodium and potassium, which are much more easily obtained than lithium [13], even though lithium-recycling technologies have been developed. Therefore, for the purpose of exploring alternative ESSs with high energy density, good electrochemical performance, and economic efficiency, abundant resources, such as sodium and potassium, are more desirable for constructing future storage systems. Recently, a series of Mg^2+^/Li^+^ [14,15], Li^+^/Na^+^ [16], and Mg^2+^/Na^+^ [17] hybrid batteries were proposed in elementary research using a hybrid-ion electrolyte. Unfortunately, relevant investigations on Na^+^/K^+^ hybrid batteries have not been performed, despite their abundant reserves. It is likely that exploring another widely used ESS without Li will bring a brighter prospect. Compared to other systems, the Na/K hybrids possess the natural advantage of low cost. Besides, the lower redox potentials (−2.93 V for K^+^/K and −2.71 V for Na^+^/Na) provide higher working voltages. In this work, a low-cost Na^+^/K^+^ hybrid battery was first established by combining the advantages of the high specific energy and abundant commercial resources of sodium and potassium with the stable cycle performance of SPAN, which has been proven to be successful in LIBs. In this novel Na^+^/K^+^ hybrid battery, SPAN nanocomposites were used as the cathode material, and elemental Na was used as the anode and current collector. Considering cost efficiency and environmental conservation, nonnoble metals and conventional carbonate electrolytes were utilized in this battery. A battery with a Na foil anode, SPAN nanocomposite cathode, and hybrid electrolyte consisting of a 0.9 mol NaPF_6_ and 0.1 mol KPF_6_ solution exhibited a remarkable electrochemical performance with outstanding reversibility of 562.2 mAh g_cathode_^−1^, i.e., 1405.5 mAh g_sulfur_^−1^ after 100 cycles, with approximately 89.8% capacity retention at a current density of 0.05 C (1 C = 700 mA g^−1^) in the voltage range of 0–3.0 V. 

## 2. Experiments

The SPAN nanocomposites were synthesized by a conventional solid-state reaction using sulfur and PAN. In total, 4.0 g of sublimed sulfur and 0.6 g of PAN were prepared and ground to create a homogeneous mixture that was then heated at 300 °C in a continuous nitrogen-filled atmosphere for 8 h. The cathode electrode was prepared with 80 wt % SPAN composite, 10 wt% acetylene black as the conductive agent, and 10 wt % poly(vinylidene fluoride) (PVDF) in N-methyl-2-pyrrolidone as the binder. The slurry was coated on Al foil to create a layer with a thickness of 200 µm and dried in a vacuum oven at 80 °C overnight. The loading amount of SPAN composites was about 1–1.2 mg·cm^−2^. The CR2032-type cells were assembled with Na foil as the anode, Celgard 2500 as the separator, and a 0.9 mol NaPF_6_ and 0.1 mol KPF_6_ solution in a mixture of ethylene carbonate (EC), dimethyl carbonate (DMC), and ethyl methyl carbonate (EMC) (4:3:2 wt%) as the electrolyte.

Electrochemical performance was tested using a LAND-CT2001A cycler (Wuhan, China) under ambient conditions. Cyclic voltammetry (CV) curves were recorded at a CHI660E (Shanghai, China) workstation. The microstructures and morphologies of the electrodes were determined by an X-ray diffractometer (XRD, Rint-2000, Rigaku, Changsha, China), a scanning electron microscope (SEM, Quanta FEG 250, FEI, Changsha, China), and energy-dispersive analysis of X-rays (EDAX, FEI, Changsha, China). For the preparation of the electrodes to be tested, tetrahydrofuran (THF) was used to wash the electrodes. Each electrode was soaked and shaken in clean THF three times and dried in Argon-filled glovebox.

## 3. Results and Discussion

**Figure 1:**
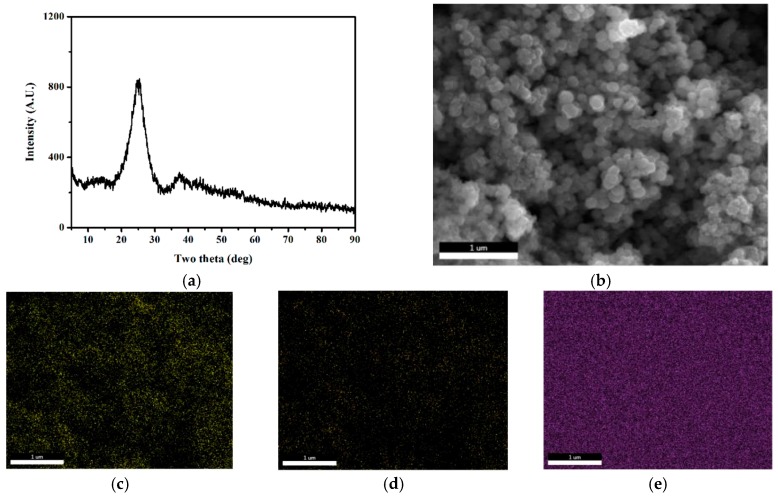
Morphology, structure characterization, and elemental analysis of the pristine sulfurized polyacrylonitrile (SPAN) nanocomposite: (**a**) XRD pattern and (**b**) FESEM image. EDAX elemental overlay of SPAN composite: (**c**) carbon, (**d**) nitrogen, and (**e**) sulfur in the sample shown in (**b**).

The SEM image (Figure 1b) shows that the average particle size of the SPAN powder was ~200 nm. The elemental overlay results obtained from EDAX illustrated that elements of carbon, nitrogen, and sulfur were distributed homogeneously in the SPAN nanocomposite (Figure 1c–e). The XRD results for SPAN (Figure 1a) highlighted that no signature was observed for S_8_. Instead, the majority of the sulfur in SPAN was present as a short chain. The short-chain sulfur was charged and discharged through a solid–solid phase transition without forming a polysulfide intermediate, which is different from the mechanism of S_8_ [18]. According to the analysis of the elemental spectrum, the active material content (sulfur) was 39.5 wt %, which was close to the experimental value from previous research [19].

**Figure 2:**
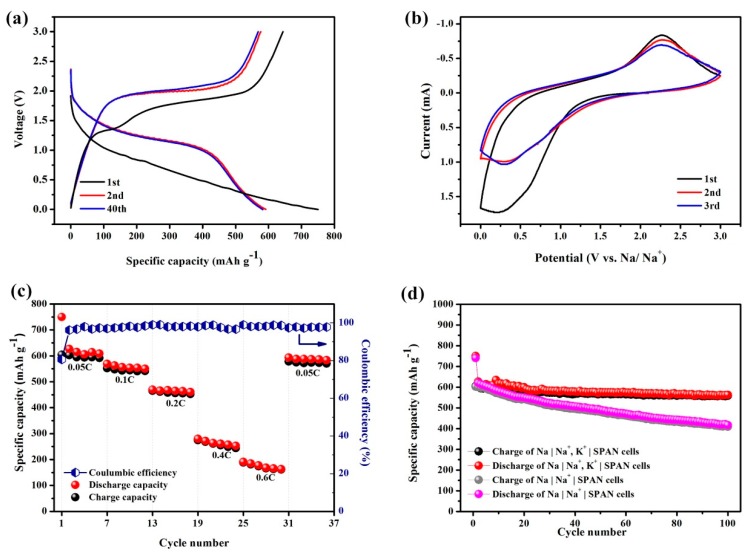
Electrochemical performance of a Na|Na^+^ and K^+^|SPAN cell. (**a**) Galvanostatic discharge–charge voltage profiles of the hybrid Na^+^/K^+^ battery at a current density of 35 mA g^−1^, (**b**) cyclic voltammetry (CV) curves at 0.5 mV s^−1^ for the first three cycles, (**c**) rate performance, and (**d**) cycle performance of Na|Na^+^, K^+^|SPAN, and Na|Na^+^|SPAN cell.

A typical cyclic galvanostatic discharge–charge curve of the hybrid Na^+^/K^+^ battery is presented in Figure 2a. During the first cycle of the discharge process, the specific capacity was measured to be 750.1 mAh g^−1^, i.e., 1875.3 mAh g_sulfur_^−1^. It is reported that there is a complicated activation process between rearranged sulfur atoms and C–S bonds during the first discharge process of a sulfur-based cathode [20], which could explain why the following cycles exhibited a lower specific capacity than the first cycle. According to Figure 2a, the voltage plateau of the first discharge (~1.0 V) was lower than that during the subsequent cycles (~1.5 V), with a longer discharge process, indicating the slow activation reaction of the first discharge process. During the first cycle, the decrease in the specific capacity and the change in the voltage plateau indicated that an irreversible transformation process might have occurred in the SPAN composite during the first discharge process. The irreversible capture of minor potassium ions and electrolyte decomposition might have resulted in the generation of a solid electrolyte interface (SEI) film during this process. After the second cycle, the Na^+^/K^+^ hybrid battery presented an excellent coulombic efficiency close to 100% with a highly reversible discharge–charge capacity of 592.3 mAh g^−1^ for up to 40 cycles.

The CV curves of the Na+/K+ hybrid battery obtained in the potential region from 0 to 3 V at 0.5 mV s^−1^ are displayed in Figure 2b. During the first cathodic process, a weak peak was observed in the potential range from 0 to 1 V. Consistent with the galvanostatic profile in Figure 2a, a lower discharge plateau (~1 V) was observed, possibly demonstrating an irreversible reaction during the discharge process of the first cycle. During the anodic process in the first three cycles, one peak was observed near 2.25 V versus Na+/Na, indicating the oxidation reaction of the discharge products while charging. Figure 2c shows the cycling performance rate of the Na^+^/K^+^ hybrid battery at different current densities. As shown in the graph, as the current density increased in a stepwise manner, the discharge–charge capacity of the Na^+^/K^+^ hybrid battery decreased in steps because the SPAN was electrochemically polarized to different extents. At current densities of 0.05 C, 0.1 C, 0.2 C, 0.4 C, and 0.6 C (1 C = 700 mAh g^−1^), the delivered discharge specific capacities were 614.3, 556.2, 465.5, 261.6, and 168.4 mAh g^−1^, respectively. Additionally, as the current density returned to the starting rate of 0.05 C in the 30th cycle, the specific discharging capacity recovered, resulting in a specific capacity of 586.5 mAh g^−1^, with a coulombic efficiency above 98%.

To verify the advantage of the addition of K in this system, a control group without addition of K was prepared with 1 M NaPF_6_ in the same carbonate solvent. As shown in Figure 2d, without the addition of K, the specific capacity decreased to 417.3 mAh g^−1^ after 100 cycles, whereas the Na|Na^+^, K^+^|SPAN cell kept a good capacity retention of 566.2 mAh g^−1^, with only 10.2% capacity loss in 100 cycles. The cycling performance of two systems, Na|Na^+^–SPAN and Na|Na^+^–K^+^|SPAN, were tested under the same current density of 35 mA g^−1^ with an equal quantity electrolyte, i.e., 10 μL/g_composite_. The difference between the two systems was possibly due to the self-healing electrostatic shield (SHES) effect of the addition of K^+^ [21]. During charging, both Na^+^ and K^+^ were adsorbed and attached on the surface of the Na anode and accumulated around the tip of Na dendrites, forming an electrostatic shield. It was determined that Na^+^/Na was −0.222 V with respect to K^+^/K. According to the simplified Nernst equation:

(1) $$E_{\mathrm{Na}}=E_{\mathrm{Na}}^{\varphi }-\frac{0.05916V}{z}\mathrm{lg}\frac{1}{c_{\mathrm{Na}}}$$

(2) $$E_{K}=E_{K}^{\varphi }-\frac{0.05916V}{z}\mathrm{lg}\frac{1}{c_{K}}$$

where *z* is the number of moles of electrons transferred, *c* is the concentration of each ions, and *E^ϕ^* is effective reduction potentials of each element. Combining Equations (1) and (2), there is a critical value of concentration ratio of K^+^/Na^+^ that make both K^+^ and Na^+^ deposit simultaneously on the anode, as expressed in Equation (3):

(3) $${\frac{c_{K}}{c_{\mathrm{Na}}}}_{critical}=10^{(E_{\mathrm{Na}}^{\varphi }-E_{K}^{\varphi })/0.05916}\approx 5656$$

As a result, in the hybrid electrolyte of 0.9 mol NaPF_6_ and 0.1 mol KPF_6_, the reduction of K^+^ on the anode did not proceed at the Na^+^ deposition potential. Moreover, this K^+^ shield prevented incoming Na^+^ from deliberately depositing on the tip of the sodium dendrite, while the dissociative K^+^ absorbed to adjacent regions of the anode until a smooth layer was eventually formed. Thus, the addition of K was found to play a significant role not only in the formation of a hybrid system but also in the suppression of dendrite growth in the Na anode.

**Figure 3:**
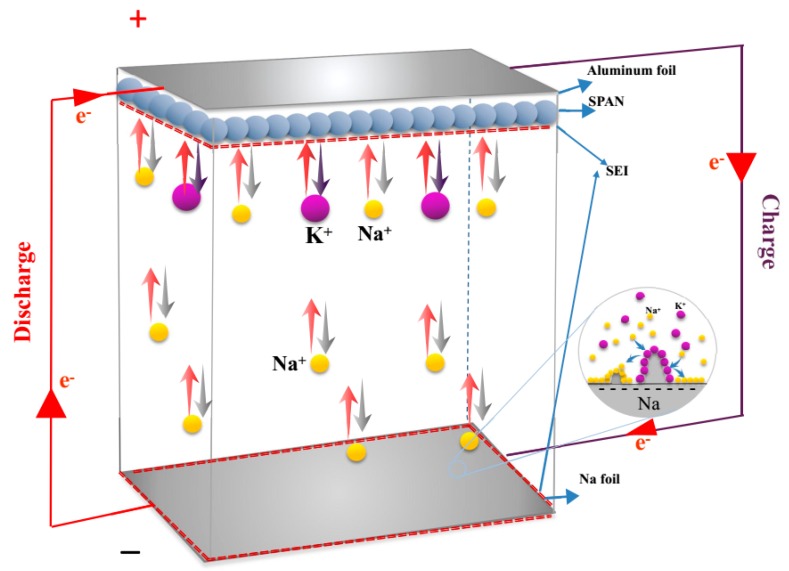
(**a**) Schematic diagram of the redox reaction in the hybrid Na^+^/K^+^ battery configuration. SEI, solid electrolyte interface.

Figure 3 shows a schematic of the discharge–charge process of the Na^+^/K^+^ hybrid battery configuration with a metallic Na foil anode, a SPAN composite cathode, and a hybrid solution. During the first discharge process, potassium ions and sodium ions transferred from the electrolyte to the SPAN composite and reacted with sulfur in the SPAN nanocomposite (Equation (4)). Simultaneously, sodium ions dissolved from the Na foil anode (Equation (5)). Conversely, while charging, the K–S and Na–S compounds disassembled, and hybrid cations migrated into the electrolyte. Meanwhile, part of sodium ions in the hybrid electrolyte deposited on the anode because of the lower standard potential (−2.93 V for K/K^+^ and −2.71 V for Na/Na^+^ vs a standard hydrogen electrode) [22].

**Figure 4:**
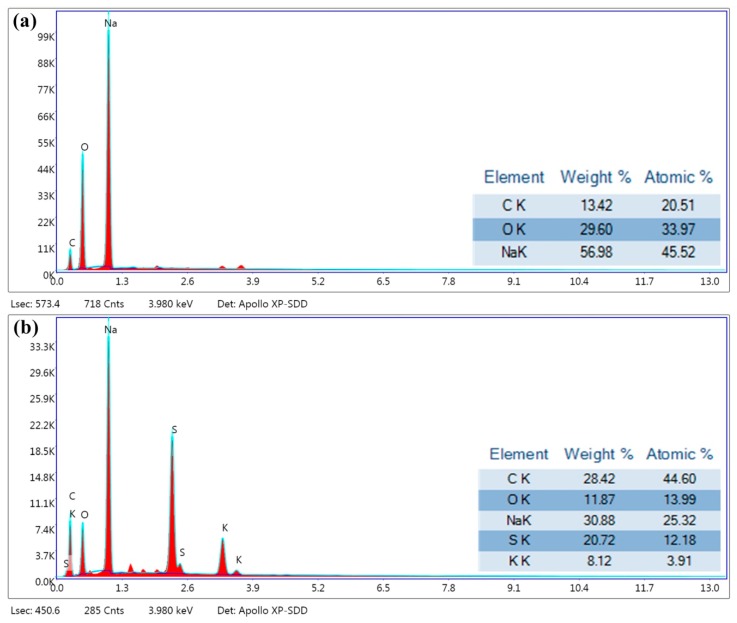
EDAX mapping results of the surface of the (**a**) charged Na anode and the (**b**) discharged SPAN composite cathode in the hybrid battery after 20 cycles.

To better understand the working mechanism of this hybrid battery, EDAX mappings of both the SPAN composite cathode and Na anode surfaces were carried out. The elemental overlay results on the Na foil (Figure 4a) showed that no K ions were found on the surface of the anode at the fully charged state of 20 cycles. The existence of C and O explains the oxidation on the surface of Na. The element mapping on the SPAN cathode was also obtained to characterize the composition process with Na^+^/K^+^ at the fully discharged state of 20 cycles; the results showed the existence of C, O, S, Na, and K (Figure 4b). The existence of Na and K could be attributed to the formation of Na–S and K–S compounds (Equation (4)), while C, O, and S are the basic elements of the oxidized SPAN composite in the atmosphere. The mass ratio of Na and K is 30.9 wt% and 8.1 wt%, which indicates Na–S and K–S composition–decomposition processes during discharging–charging, respectively. The full reaction of the system is shown as Equation (6):

(4) $$\mathrm{Cathode}:\;x{\mathrm{Na}}^{+}+yK^{+}+n{S+e}^{-}↔KyS+\mathrm{Na}xS$$

(5) $$\mathrm{Anode}:\;\mathrm{Na}↔{\mathrm{Na}}^{+}{+e}^{-}$$

(6) $$\mathrm{Full}\;\mathrm{cell}:\;\mathrm{Na}+(x-1){\mathrm{Na}}^{+}+yK^{+}+nS↔KyS+\mathrm{Na}xS$$

## 4. Conclusions

In summary, a low-cost, rechargeable Na^+^/K^+^ hybrid battery was created in this work. Using a SPAN composite as the cathode and Na foil as the anode, the battery delivered an outstanding overall performance, including a considerable specific capacity and stable cycle performance. The working mechanism of this hybrid battery was studied and verified. It involves the composition–decomposition of Na^+^ and K^+^ on the cathode and the stripping–depositing of Na^+^ at the anode with suppressed dendrites. Most importantly, on the basis of economic efficiency and remarkable electrochemical properties, this innovative Na^+^/K^+^ hybrid battery might be considered as a new flexible ESS technology with great promise. The design of hybrid batteries with a SHES technique provides inspiration for further exploration and will eventually benefit the understanding and development of next-generation electrochemical systems in the future.

## References

[1] Goodenough J.B., Kim Y. (2014). Challenges for Rechargeable Li Batteries. Chem. Mater..

[2] Amine K., Kanno R., Tzeng Y. (2014). Rechargeable lithium batteries and beyond: Progress, Challenges, and future directions. MRS Bull..

[3] Pang Q., Liang X., Kwok C.Y., Nazar L.F. (2016). Advances in lithium–sulfur batteries based on multifunctional cathodes and electrolytes. Nat. Energy.

[4] Ji X., Lee K.T., Nazar L.F. (2009). A highly ordered nanostructured carbon–sulphur cathode for lithium–sulphur batteries. Nat. Mater..

[5] Jayaprakash N., Shen J., Moganty S.S., Corona A., Archer L.A. (2011). Porous Hollow Carbon@Sulfur Composites for High-Power Lithium-Sulfur Batteries. Angew. Chem. Int. Ed..

[6] Dirlam P.T., Glass R.S., Char K., Pyun J. (2017). The use of polymers in Li-S batteries: A review. J. Polym. Sci. Part A Polym. Chem..

[7] Yang H., Naveed A., Li Q., Guo C., Chen J., Lei J., Yang J., Nuli Y., Wang J. (2018). Lithium sulfur batteries with compatible electrolyte both for stable cathode and dendrite-free anode. Energy Storage Mater..

[8] Li W., He X., Li J., Min C., Jian G., Jiang C. (2012). Charge/discharge characteristics of sulfurized polyacrylonitrile composite with different sulfur content in carbonate based electrolyte for lithium batteries. Electrochim. Acta.

[9] Yin L., Wang J., Yu X., Monroe C.W., Nuli Y., Yang J. (2012). Dual-mode sulfur-based cathode materials for rechargeable Li-S batteries. Chem. Commun..

[10] Eftekhari A., Jian Z., Ji X. (2017). Potassium Secondary Batteries. ACS Appl. Mater. Interfaces.

[11] Pramudita J.C., Sehrawat D., Goonetilleke D., Sharma N. (2017). An Initial Review of the Status of Electrode Materials for Potassium-Ion Batteries. Adv. Energy Mater..

[12] Wu X., Leonard D.P., Ji X. (2017). Emerging Non-Aqueous Potassium-Ion Batteries: Challenges and Opportunities. Chem. Mater..

[13] Taylor S.R. (1964). Abundance of chemical elements in the continental crust: A new table. Geochim. Cosmochim. Acta.

[14] Gao T., Han F., Zhu Y., Suo L., Luo C., Xu K., Wang C. (2015). Hybrid Mg^2+^/Li^+^ Battery with Long Cycle Life and High Rate Capability. Adv. Energy Mater..

[15] Fan X., Gaddam R.R., Kumar N.A., Zhao X.S. (2017). A Hybrid Mg^2+^/Li^+^ Battery Based on Interlayer-Expanded MoS_2_/Graphene Cathode. Adv. Energy Mater..

[16] Song J., Gim J., Park S., Kim J. (2018). Sodium manganese oxide electrodes accompanying self-ion exchange for lithium/sodium hybrid ion batteries. Electrochim. Acta.

[17] Li Y., An Q., Cheng Y., Liang Y., Ren Y., Sun C.J., Dong H., Tang Z., Li G., Yao Y. (2017). A high-voltage rechargeable magnesium-sodium hybrid battery. Nano Energy.

[18] Fang R., Zhao S., Sun Z., Wang D.W., Cheng H.M., Li F. (2017). More Reliable Lithium-Sulfur Batteries: Status, Solutions and Prospects. Adv. Mater..

[19] Liu Y., Wang W., Wang J., Zhang Y., Zhu Y., Chen Y., Fu L., Wu Y. (2018). Sulfur nanocomposite as a positive electrode material for rechargeable potassium-sulfur batteries. Chem. Commun..

[20] Hwang T.H., Jung D.S., Kim J.S., Kim B.G., Choi J.W. (2013). One-Dimensional Carbon–Sulfur Composite Fibers for Na–S Rechargeable Batteries Operating at Room Temperature. Nano Lett.

[21] Ding F., Xu W., Graff G.L., Zhang J., Sushko M.L., Chen X., Shao Y., Engelhard M.H., Nie Z., Xiao J. (2013). Dendrite-Free Lithium Deposition via Self-Healing Electrostatic Shield Mechanism. J. Am. Chem. Soc..

[22] Xue L., Gao H., Zhou W., Xin S., Park K., Li Y., Goodenough J.B. (2016). Liquid K–Na Alloy Anode Enables Dendrite-Free Potassium Batteries. Adv. Mater..

